# 3-(1,3-Di­phenyl­propan-2-yl)-4-methyl-6-phenyl­isoxazolo[3,4-*d*]pyridazin-7(6*H*)-one

**DOI:** 10.1107/S160053681302802X

**Published:** 2013-10-23

**Authors:** Charles F. Campana, Joseph Mirzaei, Chris Koerner, Christina Gates, Nicholas R. Natale

**Affiliations:** a Bruker AXS Inc., 5465 East Cheryl Parkway, Madison, WI 53711, USA; b The University of Montana-Missoula, The Department of Biomedical & Pharmaceutical Sciences, Missoula, MT 59812-1552, USA

## Abstract

In the title compound, C_27_H_23_N_3_O_2_, the geminal benzyl groups branching out from the methine adjacent to the isoxazole group are both *syn*-oriented to the methyl group of the pyridazinone moiety, as reflected by C—C distances of 3.812 (2) and 4.369 (2) Å between the methyl carbon and the nearest ring carbon of each benzyl group. This kind of conformation is retained in CDCl_3_ solution, as evidenced by distinct phenyl-shielding effects on the ^1^H NMR signals of the methyl H atoms. The isoxazolo[3,4-*d*]pyridazin ring system is virtually planar (r.m.s. deviation from planarity = 0.031 Å), but the N-bonded phenyl group is inclined to the former by an ring–ring angle of 55.05 (3)°. In the crystal, the T-shaped mol­ecules are arranged in an inter­locked fashion, forming rod-like assemblies along [10-1]. The mol­ecules are held together by unremarkable weak C—H⋯N, C—H⋯O and C—H⋯π inter­actions (C—O,N,C > 3.4 A), while significant π–π-stacking inter­actions are absent.

## Related literature

For chemistry of isoxazolo[3,4-*d*]pyridazinone preparation, see: Renzi & Dal Piaz (1965 ▶). For deprotonation with sodium alkoxides, see: Dal Piaz *et al.* (1975 ▶); Chimichi *et al.* (1986 ▶). For the rearrangement of the isoxazolo[3,4-*d*]pyridazinone ring system to pyrazole, see: Dal Piaz *et al.* (1985 ▶). For isoxazole lateral metalation, see: Natale & Niou (1984 ▶); Natale *et al.* (1985 ▶); Niou & Natale (1986 ▶); Schlicksupp & Natale (1987 ▶). For recent applications of lateral metalation and electrophilic quenching of isoxazoles to targets of biological inter­est, see: Nakamura *et al.* (2010 ▶); Hulubei *et al.* (2012 ▶). For a review of the lateral metalation and electrophilic quenching of isoxazoles, see: Natale & Mirzaei (1993 ▶).
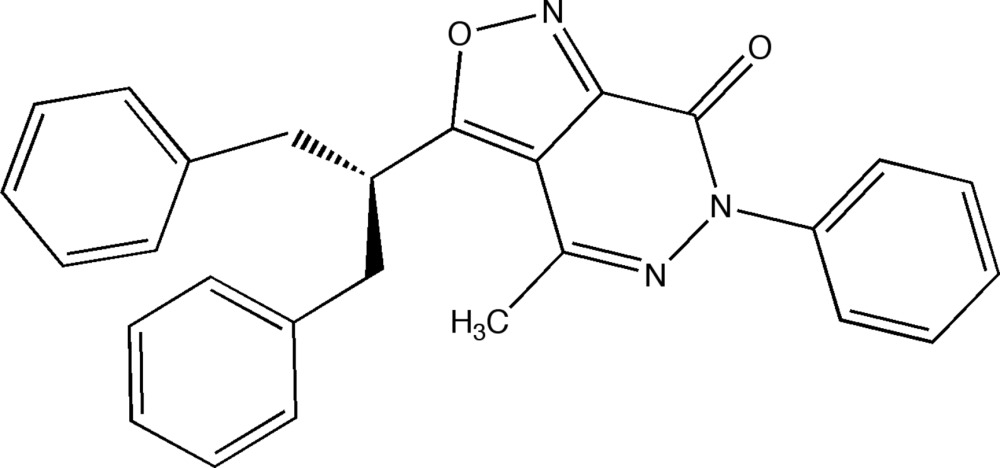


## Experimental

### 

#### Crystal data

C_27_H_23_N_3_O_2_
*M* *_r_* = 421.48
Triclinic, 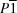

*a* = 7.5163 (4) Å
*b* = 9.6774 (5) Å
*c* = 15.9053 (8) Å
α = 86.798 (1)°
β = 83.512 (1)°
γ = 69.385 (1)°
*V* = 1075.75 (10) Å^3^
*Z* = 2
Cu *K*α radiation
μ = 0.66 mm^−1^
*T* = 100 K
0.40 × 0.22 × 0.19 mm

#### Data collection

Bruker D8 Venture PHOTON 100 CMOS diffractometer
Absorption correction: numerical (*SADABS*; Bruker, 2012 ▶) *T*_min_ = 0.80, *T*_max_ = 0.89
12012 measured reflections
3714 independent reflections
3597 reflections with *I* > 2σ(*I*)
*R*_int_ = 0.017

#### Refinement

*R*[*F* ^2^ > 2σ(*F* ^2^)] = 0.032
*wR*(*F* ^2^) = 0.078
*S* = 1.03
3714 reflections
313 parameters
86 restraints
Only H-atom displacement parameters refined
Δρ_max_ = 0.22 e Å^−3^
Δρ_min_ = −0.14 e Å^−3^

### 

Data collection: *SMART* (Bruker, 2012 ▶); cell refinement: *SAINT* (Bruker, 2012 ▶); data reduction: *SAINT*; program(s) used to solve structure: *SHELXS97* (Sheldrick, 2008 ▶); program(s) used to refine structure: *SHELXL97* (Sheldrick, 2008 ▶); molecular graphics: *Mercury* (Macrae *et al.*, 2008 ▶); software used to prepare material for publication: *publCIF* (Westrip, 2010 ▶).

## Supplementary Material

Crystal structure: contains datablock(s) global, I. DOI: 10.1107/S160053681302802X/qk2060sup1.cif

Structure factors: contains datablock(s) I. DOI: 10.1107/S160053681302802X/qk2060Isup2.hkl

Supplementary material file. DOI: 10.1107/S160053681302802X/qk2060Isup3.cml

Additional supplementary materials:  crystallographic information; 3D view; checkCIF report

## Figures and Tables

**Table 1:** Hydrogen-bond geometry (Å, °)

*D*—H⋯*A*	*D*—H	H⋯*A*	*D*⋯*A*	*D*—H⋯*A*
C26—H26⋯O1^i^	0.95	2.61	3.4159 (13)	143
C24—H24⋯N1^ii^	0.95	2.73	3.5407 (15)	143
C11—H11⋯C18^iii^	0.95	2.78	3.6182 (15)	148

Symmetry codes: (i) 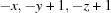; (ii) 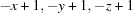; (iii) 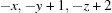.

**Fig. 1.**
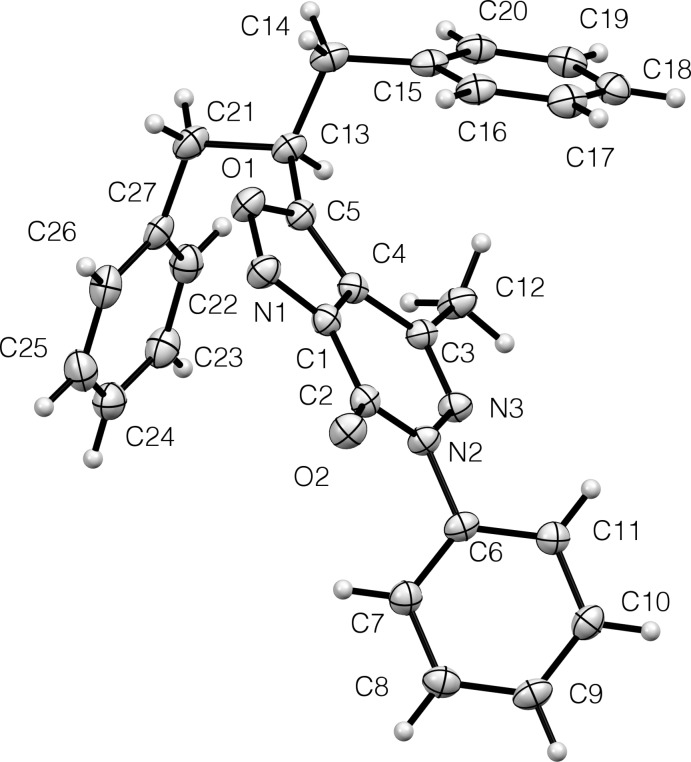
Molecular structure of the title compound, with H atoms represented by small spheres of arbitrary radius and displacement ellipsoids at the 50% probability level.

**Fig. 2.**
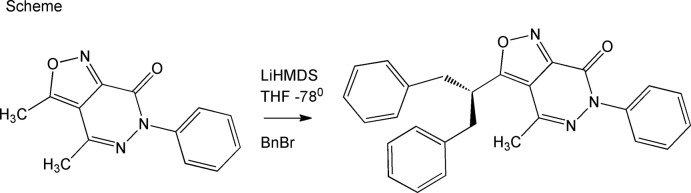
Benzylation of 3-methyl-4-methyl-6-phenylisoxazolo[3,4-d]pyridazin-7(6H)-one as precursor to give the title compound.

**Fig. 3.**
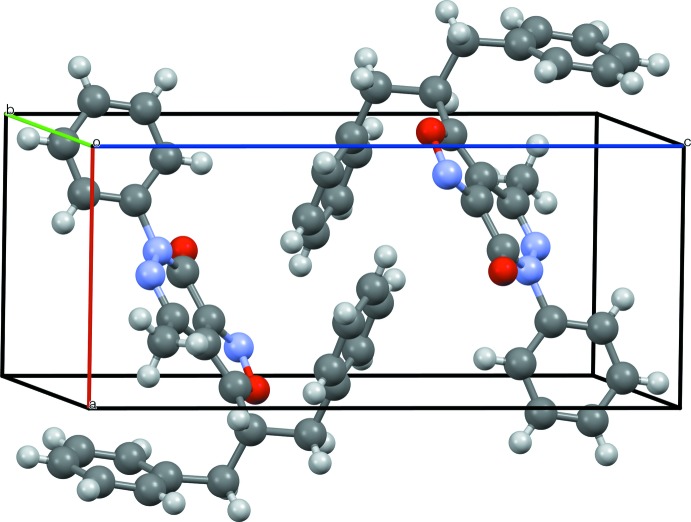
The unit cell of the title compound.

## supplementary crystallographic information

### 1. Comment

The title compound (Fig. 1) was prepared by lateral metalation with lithium hexamethyldisilazide and electrophilic quenching with benzyl bromide (Natale & Mirzaei, 1993), under thermodynamic conditions (Niou & Natale, 1986; Schlicksupp & Natale, 1987), during which a facile second deprotonation and quenching leads to double incorporation (Natale *et al.*, 1985, Natale & Niou, 1984). Mono-alkylation and recovered starting material account for sufficient material balance to rule out substantial rearrangement under these conditions. The present study unambiguously establishes the regiochemistry of double alkylation. Previous reports on analogous deprotonation with sodium alkoxides (Dal Piaz, *et al.*, 1975; Chimichi, *et al.*, 1986), reported rearrangement to pyrazoles with longer reaction times (Dal Piaz *et al.*, 1985). The lateral metalation and electrophilic quenching of isoxazoles continues to lead to candidates with promising biological activity (Nakamura, *et al.*, 2010; Hulubei *et al.*, 2012) and is the subject of active investigation, to be reported in due course. The conformation observed in the solid state (Fig. 1) would be expected to result in magnetic anisotropy if maintained in solution, and this is indeed observed, as the ^1^H NMR resonance of the C(4) methyl is observed at δ 2.55 in the starting material, δ 2.21 in the monoalkylated product, and δ 1.86 in the title compound. Further chemistry and pharmacology studies based upon this reaction are underway and will be reported in due course.

### 2. Experimental

Starting material, 3-methyl-4-methyl-6-phenylisoxazolo[3,4-d]pyridazin-7(6H)-one (Fig. 2) was prepared according to Renzi and Dal Piaz (1965). To starting material (88 mg, 0.36 mmol) was added freshly distilled tetrahydrofuran (THF, 25 ml), under an argon atmosphere. The temperature was lowered to 195 K, and a solution of lithium hexamethyldisilazide (1 ml, 1.0M in THF, Aldrich, 28% excess) was added dropwise over five minutes. After stirring for 1 h, benzyl bromide was added via syringe (0.1 ml, 0.84 mmol, 14% excess). The reaction was allowed to come to room temperature with stirring overnight, after which time the solvent was removed in vacuo by rotary evaporator, and the residue chromatographed on an 80 x 35 cm silica gel column. Gradient chromatogrpahy was performed beginning with chloroform-hexane (1:1), and the gradient slowly increased in polarity to ethyl acetate (EtOAc)-hexane-chloroform (1:2:1). The product 3-(1,3-diphenylpropan-2-yl)-4-methyl-6-phenylisoxazolo[3,4-d]pyridazin-7(6H)-one was obtained from the column fraction with R_f_ 0.6 (SiO2, EtOAc-hexane-chloroform 2:1:1) as a solid (57.1 mg, 38% yield), and was recrystallized by slow evaporation from EtOAc/hexanes to which a small amount of heptane had been added. The resulting crystals were used in the single crystal X-ray study. A clear light yellow prism-like specimen was selected for the X-ray data collection with a Bruker D8 Venture PHOTON 100 CMOS system equipped with a Cu K_α_ INCOATEC micro-focus source (λ = 1.54178 Å).

### 3. Refinement

A DELU restraint (Sheldrick, 2008) was used for the *U*_ij_ of all non-H atoms. Hydrogen atoms were positioned geometrically and refined as riding atoms, with C—H = 0.96–0.99 Å and *U*_iso_(H) = 1.5*U*_eq_(C) for methyl H atoms, and *U*_iso_(H) = 1.2*U*_eq_(C) for all other H atoms.

### Crystal data

**Table:**

C_27_H_23_N_3_O_2_	*Z* = 2
*M**_r_* = 421.48	*F*(000) = 444
Triclinic, *P*1	calculated from global refinement
Hall symbol: -P 1	*D*_x_ = 1.301 Mg m^−^^3^
*a* = 7.5163 (4) Å	Cu *K*α radiation, λ = 1.54178 Å
*b* = 9.6774 (5) Å	Cell parameters from 9923 reflections
*c* = 15.9053 (8) Å	θ = 2.8–68.4°
α = 86.798 (1)°	µ = 0.66 mm^−^^1^
β = 83.512 (1)°	*T* = 100 K
γ = 69.385 (1)°	Prism, clear light yellow
*V* = 1075.75 (10) Å^3^	0.40 × 0.22 × 0.19 mm

### Data collection

**Table:**

Bruker D8 Venture PHOTON 100 CMOS diffractometer	3714 independent reflections
Radiation source: Cu Kα	3597 reflections with *I* > 2σ(*I*)
Mirrors monochromator	*R*_int_ = 0.017
Detector resolution: 10.4167 pixels mm^-1^	θ_max_ = 66.6°, θ_min_ = 2.8°
ω and phi scans	*h* = −8→3
Absorption correction: numerical (*SADABS*; Bruker, 2012)	*k* = −11→11
*T*_min_ = 0.80, *T*_max_ = 0.89	*l* = −18→18
12012 measured reflections	

### Refinement

**Table:**

Refinement on *F*^2^	Primary atom site location: structure-invariant direct methods
Least-squares matrix: full	Secondary atom site location: difference Fourier map
*R*[*F*^2^ > 2σ(*F*^2^)] = 0.032	Hydrogen site location: inferred from neighbouring sites
*wR*(*F*^2^) = 0.078	Only H-atom displacement parameters refined
*S* = 1.03	*w* = 1/[σ^2^(*F*_o_^2^) + (0.0352*P*)^2^ + 0.337*P*] where *P* = (*F*_o_^2^ + 2*F*_c_^2^)/3
3714 reflections	(Δ/σ)_max_ < 0.001
313 parameters	Δρ_max_ = 0.22 e Å^−^^3^
86 restraints	Δρ_min_ = −0.14 e Å^−^^3^
0 constraints	

### Special details

**Table:**

Geometry. All e.s.d.'s (except the e.s.d. in the dihedral angle between two l.s. planes) are estimated using the full covariance matrix. The cell e.s.d.'s are taken into account individually in the estimation of e.s.d.'s in distances, angles and torsion angles; correlations between e.s.d.'s in cell parameters are only used when they are defined by crystal symmetry. An approximate (isotropic) treatment of cell e.s.d.'s is used for estimating e.s.d.'s involving l.s. planes.
Refinement. Refinement of *F*^2^ against ALL reflections. The weighted *R*-factor *wR* and goodness of fit *S* are based on *F*^2^, conventional *R*-factors *R* are based on *F*, with *F* set to zero for negative *F*^2^. The threshold expression of *F*^2^ > σ(*F*^2^) is used only for calculating *R*-factors(gt) *etc*. and is not relevant to the choice of reflections for refinement. *R*-factors based on *F*^2^ are statistically about twice as large as those based on *F*, and *R*- factors based on ALL data will be even larger.

### Fractional atomic coordinates and isotropic or equivalent isotropic displacement parameters (Å^2^)

**Table:**

	*x*	*y*	*z*	*U*_iso_*/*U*_eq_	
O1	0.00502 (11)	0.47448 (8)	0.64153 (5)	0.02756 (18)	
N1	0.15001 (13)	0.33590 (10)	0.65000 (6)	0.0270 (2)	
C1	0.25346 (14)	0.36020 (11)	0.70485 (6)	0.0222 (2)	
C2	0.42780 (15)	0.24940 (11)	0.73381 (6)	0.0226 (2)	
O2	0.49243 (11)	0.11970 (8)	0.71482 (5)	0.02828 (18)	
N2	0.51293 (12)	0.31237 (9)	0.78613 (5)	0.02217 (19)	
N3	0.44085 (12)	0.45480 (9)	0.81882 (5)	0.0235 (2)	
C3	0.28457 (14)	0.54911 (11)	0.79389 (6)	0.0224 (2)	
C4	0.18507 (14)	0.50724 (11)	0.73305 (6)	0.0217 (2)	
C5	0.02762 (15)	0.57547 (12)	0.69057 (6)	0.0232 (2)	
C6	0.69832 (14)	0.22965 (11)	0.81342 (7)	0.0224 (2)	
C7	0.85159 (15)	0.16976 (11)	0.75336 (7)	0.0260 (2)	
H7	0.8334	0.1771	0.6949	0.030 (3)*	
C8	1.03243 (15)	0.09880 (12)	0.77969 (7)	0.0278 (2)	
H8	1.1385	0.0572	0.739	0.034 (3)*	
C9	1.05866 (16)	0.08840 (12)	0.86484 (7)	0.0284 (2)	
H9	1.1823	0.0391	0.8826	0.033 (3)*	
C10	0.90441 (16)	0.15001 (12)	0.92425 (7)	0.0273 (2)	
H10	0.9229	0.1434	0.9827	0.032 (3)*	
C11	0.72289 (15)	0.22140 (11)	0.89891 (7)	0.0248 (2)	
H11	0.6171	0.264	0.9396	0.025 (3)*	
C12	0.21729 (16)	0.69938 (12)	0.83066 (8)	0.0295 (2)	
H12A	0.3036	0.7028	0.8717	0.039 (4)*	
H12B	0.0879	0.7216	0.8591	0.039 (4)*	
H12C	0.2161	0.7725	0.7854	0.043 (4)*	
C13	−0.11611 (15)	0.72853 (12)	0.68583 (7)	0.0255 (2)	
H13	−0.0688	0.7945	0.7162	0.019 (3)*	
C14	−0.31156 (15)	0.73629 (12)	0.73267 (7)	0.0279 (2)	
H14A	−0.4109	0.831	0.7186	0.032 (3)*	
H14B	−0.3481	0.6549	0.7141	0.029 (3)*	
C15	−0.29925 (14)	0.72404 (12)	0.82684 (7)	0.0256 (2)	
C16	−0.24282 (15)	0.58718 (12)	0.86891 (7)	0.0280 (2)	
H16	−0.222	0.5	0.8387	0.028 (3)*	
C17	−0.21682 (16)	0.57717 (13)	0.95421 (7)	0.0306 (3)	
H17	−0.1802	0.4835	0.9822	0.038 (4)*	
C18	−0.24399 (15)	0.70303 (13)	0.99886 (7)	0.0302 (3)	
H18	−0.2237	0.6958	1.0571	0.034 (3)*	
C19	−0.30114 (15)	0.83994 (13)	0.95784 (7)	0.0296 (2)	
H19	−0.3209	0.9268	0.9882	0.032 (3)*	
C20	−0.32932 (15)	0.85002 (12)	0.87290 (7)	0.0278 (2)	
H20	−0.3697	0.9442	0.8456	0.034 (3)*	
C21	−0.12730 (16)	0.78383 (13)	0.59301 (7)	0.0302 (3)	
H21A	−0.1683	0.7183	0.5602	0.029 (3)*	
H21B	−0.2235	0.8846	0.5907	0.038 (4)*	
C22	0.13242 (17)	0.89367 (13)	0.57720 (7)	0.0308 (3)	
H22	0.0532	0.9691	0.6146	0.037 (4)*	
C23	0.31273 (18)	0.89326 (13)	0.54685 (7)	0.0336 (3)	
H23	0.357	0.9668	0.5641	0.039 (4)*	
C24	0.42832 (17)	0.78538 (14)	0.49126 (7)	0.0343 (3)	
H24	0.5519	0.7848	0.47	0.039 (4)*	
C25	0.36233 (18)	0.67858 (14)	0.46702 (7)	0.0355 (3)	
H25	0.4407	0.6048	0.4285	0.046 (4)*	
C26	0.18236 (18)	0.67824 (13)	0.49847 (7)	0.0322 (3)	
H26	0.1394	0.6036	0.4817	0.036 (3)*	
C27	0.06465 (16)	0.78583 (12)	0.55409 (7)	0.0271 (2)	

### Atomic displacement parameters (Å^2^)

**Table:**

	*U*^11^	*U*^22^	*U*^33^	*U*^12^	*U*^13^	*U*^23^
O1	0.0275 (4)	0.0247 (4)	0.0302 (4)	−0.0066 (3)	−0.0104 (3)	0.0004 (3)
N1	0.0273 (5)	0.0231 (5)	0.0295 (5)	−0.0062 (4)	−0.0071 (4)	0.0001 (4)
C1	0.0238 (5)	0.0218 (5)	0.0219 (5)	−0.0093 (4)	−0.0024 (4)	0.0019 (4)
C2	0.0245 (5)	0.0203 (5)	0.0230 (5)	−0.0085 (4)	−0.0019 (4)	0.0013 (4)
O2	0.0309 (4)	0.0197 (4)	0.0334 (4)	−0.0068 (3)	−0.0064 (3)	−0.0008 (3)
N2	0.0219 (4)	0.0175 (4)	0.0257 (4)	−0.0045 (3)	−0.0044 (3)	0.0001 (3)
N3	0.0227 (4)	0.0194 (4)	0.0273 (5)	−0.0057 (3)	−0.0033 (3)	−0.0016 (3)
C3	0.0203 (5)	0.0218 (5)	0.0249 (5)	−0.0074 (4)	−0.0030 (4)	0.0017 (4)
C4	0.0218 (5)	0.0200 (5)	0.0229 (5)	−0.0075 (4)	−0.0013 (4)	0.0021 (4)
C5	0.0232 (5)	0.0241 (5)	0.0236 (5)	−0.0094 (4)	−0.0042 (4)	0.0017 (4)
C6	0.0216 (5)	0.0163 (5)	0.0296 (5)	−0.0063 (4)	−0.0059 (4)	0.0031 (4)
C7	0.0282 (6)	0.0219 (5)	0.0261 (5)	−0.0065 (4)	−0.0039 (4)	0.0013 (4)
C8	0.0244 (5)	0.0222 (5)	0.0336 (6)	−0.0045 (4)	−0.0011 (4)	−0.0012 (4)
C9	0.0241 (5)	0.0215 (5)	0.0383 (6)	−0.0049 (4)	−0.0097 (4)	0.0030 (4)
C10	0.0300 (6)	0.0238 (5)	0.0283 (6)	−0.0083 (4)	−0.0089 (4)	0.0037 (4)
C11	0.0249 (5)	0.0219 (5)	0.0273 (5)	−0.0081 (4)	−0.0027 (4)	0.0013 (4)
C12	0.0243 (5)	0.0244 (6)	0.0389 (6)	−0.0051 (4)	−0.0086 (5)	−0.0052 (5)
C13	0.0237 (5)	0.0230 (5)	0.0295 (6)	−0.0065 (4)	−0.0082 (4)	0.0033 (4)
C14	0.0224 (5)	0.0244 (6)	0.0363 (6)	−0.0064 (4)	−0.0080 (4)	0.0026 (4)
C15	0.0171 (5)	0.0251 (5)	0.0349 (6)	−0.0078 (4)	−0.0035 (4)	0.0023 (4)
C16	0.0235 (5)	0.0232 (5)	0.0387 (6)	−0.0097 (4)	−0.0046 (4)	0.0010 (5)
C17	0.0270 (6)	0.0267 (6)	0.0391 (6)	−0.0116 (4)	−0.0044 (5)	0.0081 (5)
C18	0.0246 (5)	0.0354 (6)	0.0303 (6)	−0.0111 (5)	−0.0002 (4)	0.0022 (5)
C19	0.0230 (5)	0.0274 (6)	0.0365 (6)	−0.0072 (4)	0.0013 (4)	−0.0040 (5)
C20	0.0210 (5)	0.0214 (5)	0.0384 (6)	−0.0050 (4)	−0.0018 (4)	0.0033 (4)
C21	0.0293 (6)	0.0295 (6)	0.0318 (6)	−0.0084 (5)	−0.0124 (5)	0.0076 (5)
C22	0.0362 (6)	0.0266 (6)	0.0277 (6)	−0.0081 (5)	−0.0059 (5)	0.0013 (4)
C23	0.0416 (7)	0.0345 (6)	0.0302 (6)	−0.0188 (5)	−0.0100 (5)	0.0050 (5)
C24	0.0332 (6)	0.0421 (7)	0.0279 (6)	−0.0138 (5)	−0.0056 (5)	0.0075 (5)
C25	0.0425 (7)	0.0348 (7)	0.0256 (6)	−0.0099 (5)	−0.0003 (5)	−0.0002 (5)
C26	0.0437 (7)	0.0307 (6)	0.0251 (5)	−0.0153 (5)	−0.0088 (5)	0.0018 (4)
C27	0.0304 (6)	0.0271 (6)	0.0235 (5)	−0.0081 (4)	−0.0116 (4)	0.0078 (4)

### Geometric parameters (Å, º)

**Table:**

O1—C5	1.3506 (13)	C13—H13	1.0
O1—N1	1.4100 (11)	C14—C15	1.5067 (16)
N1—C1	1.3138 (14)	C14—H14A	0.99
C1—C4	1.4122 (14)	C14—H14B	0.99
C1—C2	1.4734 (14)	C15—C20	1.3937 (16)
C2—O2	1.2176 (13)	C15—C16	1.3968 (15)
C2—N2	1.3873 (13)	C16—C17	1.3857 (17)
N2—N3	1.3979 (12)	C16—H16	0.95
N2—C6	1.4434 (13)	C17—C18	1.3851 (17)
N3—C3	1.2961 (13)	C17—H17	0.95
C3—C4	1.4425 (15)	C18—C19	1.3898 (16)
C3—C12	1.4909 (15)	C18—H18	0.95
C4—C5	1.3688 (15)	C19—C20	1.3839 (17)
C5—C13	1.4986 (14)	C19—H19	0.95
C6—C7	1.3851 (15)	C20—H20	0.95
C6—C11	1.3869 (15)	C21—C27	1.5105 (16)
C7—C8	1.3906 (16)	C21—H21A	0.99
C7—H7	0.95	C21—H21B	0.99
C8—C9	1.3835 (16)	C22—C23	1.3850 (17)
C8—H8	0.95	C22—C27	1.3940 (16)
C9—C10	1.3861 (16)	C22—H22	0.95
C9—H9	0.95	C23—C24	1.3847 (18)
C10—C11	1.3895 (15)	C23—H23	0.95
C10—H10	0.95	C24—C25	1.3825 (18)
C11—H11	0.95	C24—H24	0.95
C12—H12A	0.98	C25—C26	1.3893 (18)
C12—H12B	0.98	C25—H25	0.95
C12—H12C	0.98	C26—C27	1.3881 (16)
C13—C21	1.5431 (15)	C26—H26	0.95
C13—C14	1.5492 (15)		
			
C5—O1—N1	110.86 (8)	C14—C13—H13	107.2
C1—N1—O1	103.37 (8)	C15—C14—C13	109.92 (8)
N1—C1—C4	113.41 (9)	C15—C14—H14A	109.7
N1—C1—C2	124.88 (9)	C13—C14—H14A	109.7
C4—C1—C2	121.68 (9)	C15—C14—H14B	109.7
O2—C2—N2	123.36 (9)	C13—C14—H14B	109.7
O2—C2—C1	125.71 (10)	H14A—C14—H14B	108.2
N2—C2—C1	110.93 (9)	C20—C15—C16	118.33 (10)
C2—N2—N3	127.80 (8)	C20—C15—C14	119.92 (10)
C2—N2—C6	120.74 (8)	C16—C15—C14	121.55 (10)
N3—N2—C6	111.46 (8)	C17—C16—C15	120.73 (10)
C3—N3—N2	119.61 (9)	C17—C16—H16	119.6
N3—C3—C4	120.23 (9)	C15—C16—H16	119.6
N3—C3—C12	116.70 (9)	C18—C17—C16	120.33 (10)
C4—C3—C12	123.07 (9)	C18—C17—H17	119.8
C5—C4—C1	104.21 (9)	C16—C17—H17	119.8
C5—C4—C3	136.51 (10)	C17—C18—C19	119.48 (11)
C1—C4—C3	119.28 (9)	C17—C18—H18	120.3
O1—C5—C4	108.15 (9)	C19—C18—H18	120.3
O1—C5—C13	115.96 (9)	C20—C19—C18	120.14 (11)
C4—C5—C13	135.89 (10)	C20—C19—H19	119.9
C7—C6—C11	121.11 (10)	C18—C19—H19	119.9
C7—C6—N2	119.36 (9)	C19—C20—C15	120.97 (10)
C11—C6—N2	119.33 (9)	C19—C20—H20	119.5
C6—C7—C8	119.17 (10)	C15—C20—H20	119.5
C6—C7—H7	120.4	C27—C21—C13	110.58 (9)
C8—C7—H7	120.4	C27—C21—H21A	109.5
C9—C8—C7	120.33 (10)	C13—C21—H21A	109.5
C9—C8—H8	119.8	C27—C21—H21B	109.5
C7—C8—H8	119.8	C13—C21—H21B	109.5
C8—C9—C10	119.94 (10)	H21A—C21—H21B	108.1
C8—C9—H9	120.0	C23—C22—C27	121.42 (11)
C10—C9—H9	120.0	C23—C22—H22	119.3
C9—C10—C11	120.40 (10)	C27—C22—H22	119.3
C9—C10—H10	119.8	C24—C23—C22	119.85 (11)
C11—C10—H10	119.8	C24—C23—H23	120.1
C6—C11—C10	119.05 (10)	C22—C23—H23	120.1
C6—C11—H11	120.5	C25—C24—C23	119.42 (11)
C10—C11—H11	120.5	C25—C24—H24	120.3
C3—C12—H12A	109.5	C23—C24—H24	120.3
C3—C12—H12B	109.5	C24—C25—C26	120.56 (11)
H12A—C12—H12B	109.5	C24—C25—H25	119.7
C3—C12—H12C	109.5	C26—C25—H25	119.7
H12A—C12—H12C	109.5	C27—C26—C25	120.70 (11)
H12B—C12—H12C	109.5	C27—C26—H26	119.6
C5—C13—C21	110.34 (9)	C25—C26—H26	119.6
C5—C13—C14	110.96 (9)	C26—C27—C22	118.05 (11)
C21—C13—C14	113.70 (9)	C26—C27—C21	121.96 (10)
C5—C13—H13	107.2	C22—C27—C21	119.91 (10)
C21—C13—H13	107.2		
			
C5—O1—N1—C1	0.42 (11)	C6—C7—C8—C9	−0.07 (16)
O1—N1—C1—C4	−0.08 (11)	C7—C8—C9—C10	−0.54 (16)
O1—N1—C1—C2	−178.31 (9)	C8—C9—C10—C11	0.47 (16)
N1—C1—C2—O2	−4.53 (17)	C7—C6—C11—C10	−0.83 (16)
C4—C1—C2—O2	177.38 (10)	N2—C6—C11—C10	−175.73 (9)
N1—C1—C2—N2	174.91 (10)	C9—C10—C11—C6	0.20 (16)
C4—C1—C2—N2	−3.18 (13)	O1—C5—C13—C21	−53.31 (12)
O2—C2—N2—N3	−172.66 (9)	C4—C5—C13—C21	125.98 (13)
C1—C2—N2—N3	7.88 (14)	O1—C5—C13—C14	73.63 (11)
O2—C2—N2—C6	7.42 (15)	C4—C5—C13—C14	−107.07 (14)
C1—C2—N2—C6	−172.03 (8)	C5—C13—C14—C15	71.49 (11)
C2—N2—N3—C3	−6.84 (15)	C21—C13—C14—C15	−163.44 (9)
C6—N2—N3—C3	173.09 (9)	C13—C14—C15—C20	86.05 (12)
N2—N3—C3—C4	0.28 (14)	C13—C14—C15—C16	−88.63 (12)
N2—N3—C3—C12	−179.32 (9)	C20—C15—C16—C17	−0.22 (15)
N1—C1—C4—C5	−0.26 (12)	C14—C15—C16—C17	174.54 (10)
C2—C1—C4—C5	178.03 (9)	C15—C16—C17—C18	−0.90 (16)
N1—C1—C4—C3	179.63 (9)	C16—C17—C18—C19	1.23 (16)
C2—C1—C4—C3	−2.08 (15)	C17—C18—C19—C20	−0.44 (16)
N3—C3—C4—C5	−176.42 (11)	C18—C19—C20—C15	−0.69 (16)
C12—C3—C4—C5	3.15 (19)	C16—C15—C20—C19	1.01 (15)
N3—C3—C4—C1	3.73 (15)	C14—C15—C20—C19	−173.84 (10)
C12—C3—C4—C1	−176.70 (10)	C5—C13—C21—C27	−59.69 (12)
N1—O1—C5—C4	−0.60 (11)	C14—C13—C21—C27	174.90 (9)
N1—O1—C5—C13	178.88 (8)	C27—C22—C23—C24	1.06 (17)
C1—C4—C5—O1	0.51 (11)	C22—C23—C24—C25	−0.34 (17)
C3—C4—C5—O1	−179.35 (11)	C23—C24—C25—C26	−0.50 (17)
C1—C4—C5—C13	−178.82 (11)	C24—C25—C26—C27	0.65 (17)
C3—C4—C5—C13	1.3 (2)	C25—C26—C27—C22	0.05 (16)
C2—N2—C6—C7	56.43 (13)	C25—C26—C27—C21	−176.67 (10)
N3—N2—C6—C7	−123.50 (10)	C23—C22—C27—C26	−0.90 (16)
C2—N2—C6—C11	−128.58 (10)	C23—C22—C27—C21	175.88 (10)
N3—N2—C6—C11	51.49 (12)	C13—C21—C27—C26	103.38 (12)
C11—C6—C7—C8	0.76 (16)	C13—C21—C27—C22	−73.27 (12)
N2—C6—C7—C8	175.67 (9)		

### Hydrogen-bond geometry (Å, º)

**Table:**

*D*—H···*A*	*D*—H	H···*A*	*D*···*A*	*D*—H···*A*
C26—H26···O1^i^	0.95	2.61	3.4159 (13)	143
C24—H24···N1^ii^	0.95	2.73	3.5407 (15)	143
C11—H11···C18^iii^	0.95	2.78	3.6182 (15)	148

Symmetry codes: (i) −*x*, −*y*+1, −*z*+1; (ii) −*x*+1, −*y*+1, −*z*+1; (iii) −*x*, −*y*+1, −*z*+2.
